# Anti-glomerular basement membrane disease associated with thin basement membrane nephropathy: A case report

**DOI:** 10.1097/MD.0000000000026095

**Published:** 2021-05-21

**Authors:** Chi Young Jung, Sun-Jae Lee, Min-Kyung Kim, Dong Jik Ahn, In Hee Lee

**Affiliations:** a Department of Internal Medicine; b Department of Pathology, Daegu Catholic University School of Medicine, Daegu; c Department of Pathology, Dongguk University College of Medicine, Gyeongju; d Department of Internal Medicine, HANSUNG Union Internal Medicine Clinic and Dialysis Center, Daegu, Republic of Korea.

**Keywords:** anti-glomerular basement membrane disease, plasmapheresis, pulmonary hemorrhage, thin basement membrane nephropathy

## Abstract

**Rationale:**

Simultaneous occurrence of anti-glomerular basement membrane (anti-GBM) disease and thin basement membrane nephropathy (TBMN), both of which invade the type IV collagen subunits, is very rare. Here, we present the case of a 20-year-old male patient diagnosed with both anti-GBM disease and TBMN upon presenting dyspnea and hemoptysis.

**Patient concerns:**

No laboratory abnormalities, except arterial hypoxemia (PaO_2_75.4 mmHg) and microscopic hematuria, were present. Chest computed tomography revealed bilateral infiltrations in the lower lung fields; thus, administration of empirical antibiotics was initiated. Gross hemoptysis persisted nonetheless, and bronchoscopy revealed diffuse pulmonary hemorrhage with no endobronchial lesions. Broncho-alveolar lavage excluded bacterial pneumonia, tuberculosis, and fungal infection.

**Diagnosis:**

Enzyme-linked immunosorbent assay of his serum was positive for anti-GBM antibody (95.1 U/mL). Human leukocyte antigen (HLA) test was positive for both HLA-DR15/-DR04. Other than diffuse thinning of the GBM (average thickness, 220 nm), index kidney biopsy did not demonstrate any specific abnormalities such as crescent formation.

**Interventions:**

Methylprednisolone was administered intravenously for 7 consecutive days (500 mg/day), followed by the daily dose of oral prednisolone (80 mg). Cyclophosphamide was also orally administered every day for 3 months (250 mg/day). Following 6 sessions of plasmapheresis, the anti-GBM antibody in serum became negative.

**Outcomes:**

There was no clinical evidence suggesting recurrence of pulmonary hemorrhage or azotemia during hospitalization and 12-month follow-up period. Twelve months after hospital discharge, oral prednisolone was discontinued.

**Lessons:**

The patients with concurrent anti-GBM disease and TBMN will have a favorable prognosis after proper therapy. However, further research is needed to elucidate the pathogenesis and long-term outcome of the comorbidity of these 2 diseases.

## 1 Introduction

Anti-glomerular basement membrane (anti-GBM) disease is a rare autoimmune disease in which autoantibodies are produced against the alveolar and GBMs, inducing pulmonary capillaritis and crescentic glomerulonephritis, respectively.^[1]^ Diffuse alveolar hemorrhage caused by pulmonary vascular infiltration can lead to life-threatening respiratory failure.^[1]^ Moreover, anti-GBM disease is observed in 1%–5% of all glomerular diseases; it is especially prevalent in rapidly progressive glomerulonephritis (RPGN) at 10%–20%.^[2]^ Thin basement membrane nephropathy (TBMN) is an inheritable or sporadic glomerulopathy in which the α3 and α4 chains of type IV collagen subunits present in the GBM are reduced owing to genetic mutations in the *COL4A3* or *COL4A4* loci.^[3]^ The thinning of GBM may be reduced to below 250 nm, as noted on electron microscopy, and may be accompanied by nonspecific glomerular lesions.^[4]^ This disease is found in 1% of the general population. In most patients, persistent isolated microscopic hematuria is the most common clinical presentation.^[5]^ Although both anti-GBM disease and TBMN affect the type IV collagen subunits in the basement membrane, the concurrent presence of these 2 diseases is very rare.^[6,7]^ The authors report the case of a 20-year-old male patient who complained of acute dyspnea accompanied by blood-tinged sputum and was diagnosed with anti-GBM disease and TBMN upon visiting the clinic.

## 2 Case presentation

**Figure 1:**
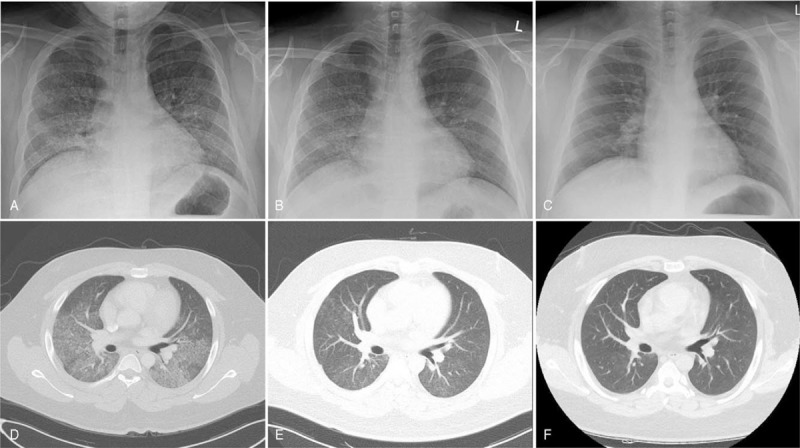
(A, D) On admission, chest radiographs showed diffuse opacities in both lung fields, and chest CT scans showed multiple small nodular and ground-glass opacities in both lung fields. (B, D) Ten days after completion of methylprednisolone pulse therapy, interval improvement of pulmonary lesions was seen. (C, E) Two months after hospital discharge, chest radiograph and CT scans showed complete resolution of previous lesions. CT = computed tomography.

**Figure 2:**
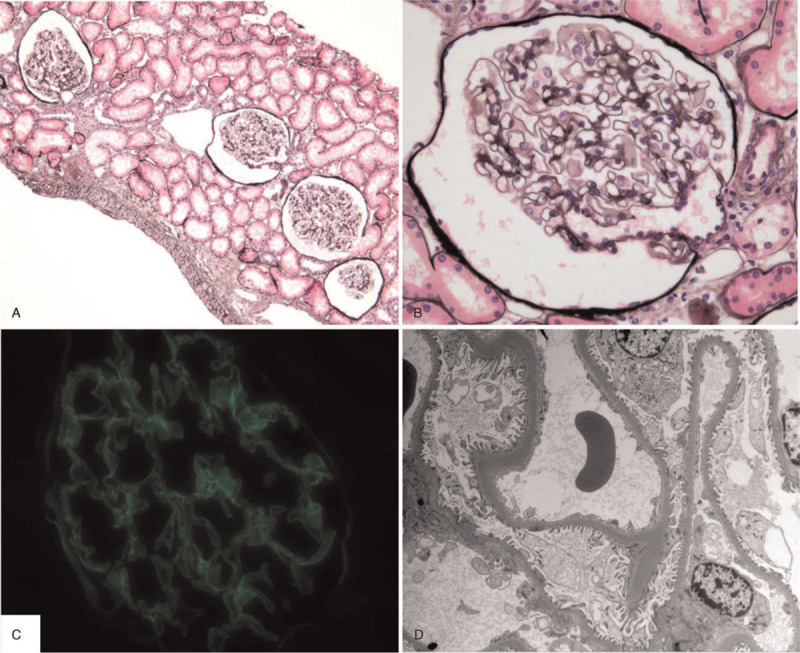
Microscopic features of renal biopsy. (A, B) The glomeruli are unremarkable without crescents and proliferation, and there is no evidence of tubulointerstitial inflammation or fibrosis (A: methenamine silver stain, ×100), (B: methenamine silver stain, ×400). (C) The GBM staining for IgG is negative (anti-IgG immunofluorescence, ×400). (D) Glomerular architecture is unremarkable other than diffuse thinning of the glomerular basement membranes, and there are no electron-dense deposits or GBM breaks (transmission electron microscopy, ×3500). IgG = immunoglobulin G, GBM = glomerular basement membrane.

A 20-year-old male patient was admitted to our hospital complaining of dyspnea that persisted for 2 weeks (modified Medical Research Council dyspnea scale, 3).^[8]^ Two weeks prior, a cough accompanied by blood-tinged sputum was observed, along with exertional dyspnea. Upon his visit to the pulmonary clinic, the patient demonstrated a blood pressure of 150/100 mmHg, pulse rate of 102 beats/min, respiration rate of 20 breaths/min, and a body temperature of 36.8°C. His past medical history was free of tuberculosis, diabetes mellitus, hypertension, and renal diseases, aside from an obesity-related fatty liver. His family history was also free of kidney disease, including microscopic hematuria. The patient had a smoking history of 1 pack per year. Chest auscultation revealed reduced breathing sounds in both lung bases without inspiratory crackles. Physical examination revealed that the patient had no pretibial edema in the lower extremities or hepatosplenomegaly. Results of a peripheral blood test conducted on admission revealed counts of 9900/μL white blood cells (WBC, 74.2% neutrophils), 14.1 g/dL hemoglobin, and 364,000/μL platelets. Serum biochemistry test results were as follows: 9.9 mg/dL blood urea nitrogen (BUN), 0.8 mg/dL creatinine (Cr), 87 IU/L aspartate aminotransferase, 192 IU/L alanine aminotransferase, 7.5 g/dL total protein, 4.3 g/dL albumin, and 32.6 mg/L C-reactive protein (reference range, <5 mg/L). Results of arterial blood gas analysis performed in normal room air conditions were pH 7.461, PaCO_2_ 32.1 mmHg, PaO_2_ 75.4 mmHg, and 96.4% oxygen saturation. Urinalysis results were pH 5.5, 1+ occult blood, and trace albumin, and urine sediment examination revealed WBCs at 1–4/high-power field (HPF) and red blood cells (RBCs) at 5–10/HPF (dysmorphic 80%). The 24-hour urine volume was 1500 mL, with the urinary protein excretion level being 242.5 mg/day and Cr clearance being 95 mL/min/1.73m^2^. Blood coagulation profiles (prothrombin time, activated partial thromboplastin time, and fibrinogen) were all found to be within the normal range. Serum immunoglobulin (Ig) G, IgA, and IgM levels were all normal. Serum complement (C) 3 level was low at 86.2 mg/dL (reference range, 90–180 mg/dL), whereas C4 and CH50 levels were normal. Furthermore, serological examinations of rheumatoid factor and viral markers (hepatitis B surface antigen, anti-hepatitis C antibody, and anti-human immunodeficiency virus antibody) were all negative. Bilateral infiltrative lesions suspected of atypical bacterial pneumonia were detected in the lower lung fields through chest radiograph and chest computed tomography (CT), after which intravenous administration of antibiotics (ceftriaxone, levofloxacin) was initiated (Fig. 1A, D). However, gross hemoptysis persisted, and bronchoscopy was performed on the 3rd day of hospitalization, which revealed the presence of diffuse hemorrhage with no endobronchial lesions. The flexible bronchoscope was wedged into the lateral segment of the right middle lobe bronchus. Sequential broncho-alveolar lavage (BAL) showed progressively more hemorrhagic effluent. Analysis of BAL fluid revealed a CD4/CD8 ratio of 0.62 (reference range, 069–2.83), RBC levels of 578,470/μL, and WBC levels of 1500/μL. Bronchial secretion cultures were negative for bacteria, mycobacteria, and fungi. Transbronchial lung biopsies were not attempted because of the probable risk of profuse bleeding and respiratory distress. Nevertheless, an enzyme-linked immunosorbent assay (ELISA) of his serum revealed an anti-GBM antibody titer of 95.1 U/mL (reference range, <20 U/mL). The results of serologic tests for antinuclear antibody, anti-neutrophil cytoplasmic antibody, and cryoglobulin were all negative, excluding connective tissue disease and systemic vasculitis. Taken together, the patient was subsequently diagnosed with anti-GBM disease. Therefore, the authors opted for treatment with 500 mg intravenous methylprednisolone for 7 consecutive days, followed by daily administration of 80 mg oral prednisolone. Cyclophosphamide was also orally administered once a day (250 mg/day). Plasmapheresis was started on the 7th day of hospitalization due to continued pulmonary hemorrhage. Plasmapheresis was performed once a day for 6 days, after which there was a reduction in the serum level of anti-GBM antibody (2.2 U/mL). Ten days after completion of corticosteroid pulse therapy, interval improvement of pulmonary lesions was noted on the radiologic examinations (Fig. 1B, E). A human leukocyte antigen (HLA) test revealed the presence of both HLA-DR15 and HLA-DR04, which are linked to genetic susceptibility for anti-GBM disease. The size and shape of the kidneys appeared normal during abdominal ultrasonography. Fourteen days after admission, a percutaneous renal biopsy, under ultrasound guidance, was performed to assess glomerular abnormalities related to the presence of anti-GBM antibody. Light microscopic examination on a total of 13 glomeruli showed no pathological abnormalities, including lack of intravascular thrombosis and formed crescents. Furthermore, there were no indications of tubular atrophy, interstitial fibrosis, or inflammatory cell infiltrations (Fig. 2A, B). Immunofluorescence microscopy did not indicate immune deposits for immunoglobulins or complements (Fig. 2C). However, electron microscopy revealed diffuse thinning of the GBM to an average thickness of 220 nm; thus, a diagnosis of TBMN was made (Fig. 2D). No heterozygous mutations in the *COL4A3* or *COL4A4* genes were identified in the genotypic analysis. The patient was discharged with a marked resolution of respiratory symptoms after 32 days in the hospital. Two months after hospital discharge, follow-up chest radiograph and chest CT scans demonstrated complete recovery of the previous pulmonary lesions (Fig. 1C, F), and oral cyclophosphamide was discontinued. Currently, 12 months after discharge, a tapered dose of oral prednisolone (10 mg/day) was withdrawn without hemoptysis. His renal function was normal at BUN 19.4 mg/dL and serum Cr 0.9 mg/dL. Moreover, there were no notable changes in urinalysis with albumin 1+, RBC 5–10/HPF, and spot urine protein/Cr of 0.215 g/g, when compared to the initial findings at the time of kidney biopsy.

## 3 Discussion

Anti-GBM disease is an autoimmune small-vessel vasculitis in which there is an abnormal production of autoantibodies against the non-collagenous (NC) 1 domain of the type IV collagen α3 chain, which is expressed in pulmonary and glomerular capillary basement membranes.^[1,9]^ The typical clinical manifestations of anti-GBM disease include subacute kidney injury with pulmonary hemorrhage (also known as pulmonary-renal syndrome). In cases complicated by RPGN, the crescent formation is accompanied by necrotizing and/or inflammatory lesions. This has been demonstrated through smooth and linear immunostaining for IgG and C3 along the GBM in the kidney biopsy specimens.^[2]^ However, pulmonary-dominant atypical anti-GBM disease, in which only linear immune deposits are observed along the basement membrane, has also been reported at a frequency under 10% in the absence of clinical evidence such as crescentic glomerulonephritis and renal failure.^[2]^

The occurrence of anti-GBM disease has been attributed to an abnormal immune response in genetically predisposed individuals, exacerbated by environmental constituents. Environmental precipitating factors include exposure to hydrocarbons, nasal inhalation of recreational drugs such as cocaine and amphetamine, smoking, and infections.^[10]^ Furthermore, HLA class II alleles, including the HLA-DRB1∗1501 and -DR4 alleles, have been demonstrated to have a consistent correlation to the susceptibility of patients developing anti-GBM disease.^[11]^ In contrast, HLA-DR1, -DR7, and -DR9 are inversely related to the development of anti-GBM disease.^[12]^ This patient had an evident history of smoking, albeit over a short period of time, and carried HLA-DR15 and HLA-DR04, which likely contributed to the occurrence of anti-GBM disease. The diagnosis of anti-GBM disease includes the detection of pathognomonic anti-GBM antibody via serology or in tissue biopsy, as well as the presence of glomerulonephritis or alveolar hemorrhage.^[10]^ ELISA, used for the diagnosis and follow-up for this patient, are reliable diagnostic approaches for counting anti-GBM antibodies circulating in the bloodstream, with high sensitivity (>95%) and specificity (>97%).^[1]^ Unlike what was observed in this case, immunofluorescence microscopy can reveal strong linear staining for IgG and C3 along the GBM even though there was a negative test for circulating anti-GBM antibody. There has also been a previous report of crescentic glomerulonephritis in the renal biopsy of a patient with anti-GBM disease who initially had normal renal function with no pulmonary hemorrhage and whose condition progressed to end-stage renal disease within 27 months despite having appropriate treatment.^[13]^ Thus, the presence of pre-existing glomerular disease should be verified even in patients with anti-GBM disease who have normal kidney function. Moreover, an index kidney biopsy should be positively considered for an accurate prediction of renal outcome.

The standard treatment for anti-GBM disease is a combination therapy consisting of the administration of cytotoxic agents such as cyclophosphamide and high-dose corticosteroids, and plasmapheresis.^[10]^ The administration of cytotoxic agents and corticosteroids inhibits the production of autoantibodies and minimizes inflammatory reactions and damage to target organs. Plasmapheresis enables rapid removal of circulating anti-GBM antibodies and other inflammatory mediators such as complements. The optimal duration of plasmapheresis and administration of immunosuppressive agents has not yet been established, although it is recommended that plasmapheresis be repeated until the circulating antibodies are eliminated.^[10]^

Thus far, there have been 3 cases in which both anti-GBM disease and TBMN were simultaneously identified (Table 1). The male to female ratio was 2:1, and the age of onset varied from 30s to 50s. Microscopic hematuria was the chief complaint in all patients, and this was accompanied by hemoptysis and dyspnea in 1 patient. Two patients had a history of smoking. The renal functions of all the patients were within the normal range. There were no consistent patterns in the degree of proteinuria and RBC casts according to urinalysis. There were no evident findings of cellular or fibro-cellular crescents in renal biopsy specimens. Nevertheless, linear immunofluorescent staining along the basement membrane, which is a classic characteristic of potential anti-GBM disease, was detected in all 3 cases. Electron microscopy revealed a significantly thinner GBM (average thickness, 220–295 nm) compared with that in healthy adults (350–450 nm). In 1 of the cases, a 37-year-old woman with dyspnea and hemoptysis, both pulmonary lesions and respiratory symptoms improved markedly following several sessions of plasmapheresis and administration of steroids.^[7]^ There were no cases demonstrating recurrence of respiratory symptoms or renal dysfunction in the follow-up period. In this case, our male patient was younger than the patients in previous cases and had a much-reduced smoking habit, of just 1 pack per year. The patient had dyspnea due to alveolar hemorrhage but did not present overt symptoms of a lower respiratory tract disease nor did he display substantial anatomic abnormalities of the bronchi. Nevertheless, anti-GBM disease was suspected owing to the high titer of anti-GBM antibody in his serum. His respiratory symptoms such as cough, blood-tinged sputum, and exertional dyspnea improved soon after standard therapy based on previous guidelines.^[10]^ The kidney biopsy demonstrated no significant pathology aside from diffuse thinning of the GBM. Furthermore, during the 12-month follow-up, there were no episodes of hemoptysis or renal impairment. Considering both previous case reports and the clinical course of this patient, it is likely that patients with concurrent anti-GBM disease and TBMN will have a favorable prognosis after proper treatment. In patients presenting with active pulmonary hemorrhage, prompt plasmapheresis could be considered as a part of essential therapy.

**Table 1:** Cases of anti-glomerular basement membrane disease in patients with thin basement membrane nephropathy.

No.	1^[6]^	2^[6]^	3^[7]^	Current case
Year reported	1990	1990	2018	2021
Age (years)/gender	55/Male	49/Male	37/Female	20/Male
Smoking history	–	+	+	+
Family history	–	–	–	–
Clinical presentation	Hematuria	Hematuria	Hematuria, dyspnea	Dyspnea, hemoptysis
Hemoptysis	–	–	+	+
Serum creatinine (mg/dL)	0.9	1.2	0.76	0.8
Serum anti-GBM antibody	IgA on RIA	Negative on RIA	Positive	Positive
Proteinuria (mg/day)	<200	<200	Not reported	242.5
Urine RBC cast	–	–	+	+
Renal pathology				
Light microscopy	Nil	Mesangial proliferative glomerulonephritis	Segmental fibrinoid necrosis	Nil
Linear IF staining along GBM	IgA	IgM, C3	IgG, C3, kappa, lambda	Nil
GBM thickness (nm)	220	295	237.7	220
Treatment	None	None	CS + PP	CS + CYP + PP
Renal function on follow-up	Normal	Normal	Normal	Normal

The underlying pathogenic mechanism of the comorbidity of a type of small vessel vasculitis, anti-GBM disease, and TBMN is not yet clear. Although it is likely that TBMN preceded the anti-GBM disease, it is nevertheless difficult to rule out coincidental comorbidity. Thinning of the GBM reduces the amount of α3 NC1 located in the basement membrane, which leads to reduced expression of anti-GBM target epitopes, thus alleviating the activity of glomerulonephritis.^[14]^ This inference can act as theoretical evidence supporting the maintenance of normal renal function in patients with comorbidities of these diseases. However, there is also potential that the exposure of the Goodpasture antigen, due to toxic or infectious insults to a thin GBM, may facilitate the structural modification of the antigen, thus triggering subsequent development of non-specific immune responses.^[6]^ Therefore, further studies are needed on the interactive mechanisms of the 2 diseases.

In summary, the authors present a case of a patient who was simultaneously diagnosed with anti-GBM disease and TBMN upon admission for dyspnea and hemoptysis. Standard therapy for anti-GBM disease, including immunosuppression and plasmapheresis, was performed, and thus, potentially fatal respiratory symptoms such as active pulmonary hemorrhage significantly improved. Moreover, there were no observations of recurrent hemoptysis or clinical evidence of azotemia throughout the 12-month follow-up. The findings from this case suggest that patients with concurrent anti-GBM disease and TBMN will have a favorable prognosis after proper therapy. However, further research may be needed on the pathogenic mechanisms of comorbidity and long-term outcomes of these 2 diseases.

## Author contributions

**Conceptualization:** In Hee Lee.

**Data curation:** Chi Young Jung, Sun-Jae Lee, In Hee Lee.

**Formal analysis:** Chi Young Jung, Min-Kyung Kim, In Hee Lee.

**Methodology:** Chi Young Jung, Dong Jik Ahn, In Hee Lee.

**Validation:** Min-Kyung Kim, Dong Jik Ahn.

**Writing – original draft:** Chi Young Jung, In Hee Lee.

**Writing – review & editing:** In Hee Lee.
